# NLM-HS: Navigation Learning Model Based on a Hippocampal–Striatal Circuit for Explaining Navigation Mechanisms in Animal Brains

**DOI:** 10.3390/brainsci11060803

**Published:** 2021-06-17

**Authors:** Jie Chai, Xiaogang Ruan, Jing Huang

**Affiliations:** 1 Faculty of Information Technology, Beijing University of Technology, Beijing 100124, China; chaijie@emails.bjut.edu.cn (J.C.); adrxg@bjut.edu.cn (X.R.); 2 Beijing Key Laboratory of Computational Intelligence and Intelligent System, Beijing 100124, China

**Keywords:** navigation, goal-directed learning, habitual learning, hippocampus, striatum

## Abstract

Neurophysiological studies have shown that the hippocampus, striatum, and prefrontal cortex play different roles in animal navigation, but it is still less clear how these structures work together. In this paper, we establish a navigation learning model based on the hippocampal–striatal circuit (NLM-HS), which provides a possible explanation for the navigation mechanism in the animal brain. The hippocampal model generates a cognitive map of the environment and performs goal-directed navigation by using a place cell sequence planning algorithm. The striatal model performs reward-related habitual navigation by using the classic temporal difference learning algorithm. Since the two models may produce inconsistent behavioral decisions, the prefrontal cortex model chooses the most appropriate strategies by using a strategy arbitration mechanism. The cognitive and learning mechanism of the NLM-HS works in two stages of exploration and navigation. First, the agent uses a hippocampal model to construct the cognitive map of the unknown environment. Then, the agent uses the strategy arbitration mechanism in the prefrontal cortex model to directly decide which strategy to choose. To test the validity of the NLM-HS, the classical Tolman detour experiment was reproduced. The results show that the NLM-HS not only makes agents show environmental cognition and navigation behavior similar to animals, but also makes behavioral decisions faster and achieves better adaptivity than hippocampal or striatal models alone.

## 1. Introduction

Spatial cognition and navigation are basic abilities that autonomous mobile robots need to possess [1,2,3]. However, most of the existing robots do not have the ability to learn, and their cognition and adaptability to unknown environments are weak. Most animals, such as rats, bats, and birds, have better environmental cognitive and adaptive abilities and can navigate to a destination effectively [4,5]. Inspired by animal navigation, bionic environmental cognition and navigation algorithms have been gradually applied to autonomous mobile robots, which not only aims to make agents more bionic and more effective in environmental cognition and navigation, but also aims to explore navigation mechanisms in the animal brain.

The nature of spatial navigation has always been a hot topic of research. Physiological studies have shown that navigation-related structures in the brain mainly include the hippocampus, striatum, and prefrontal cortex [6]. These brain regions interact to achieve navigation. A growing number of studies indicate that the hippocampal–striatal circuit is of great significance to goal-directed navigation [7,8]. The hippocampus plays a central role in spatial representation and episodic memory [9]. Tolman found that rats can freely explore and learn the layout of a maze without reinforcement factors, and proposed the concept of a cognitive map for the first time, pointing out that rats can use an internal expression of the spatial environment to recognize and remember the environment [10]. Then, O’Keefe and Dostrovsky discovered place cells in the hippocampus that can respond to specific locations [11]. The place cells can encode the position of the rats in real time, thereby generating a topological expression of the spatial environment in the brain [12]. In addition, physiological studies have shown that the rat hippocampus generates brief sequences encoding spatial trajectories from the current location to a goal location before goal-directed navigation [13,14]. These sequences are capable of predicting immediate future behavior, supporting goal-directed actions, and controlling subsequent navigational behavior [15]. Recent physiological studies suggest that anticipatory firing is also found in the striatum [16], which is part of the basal ganglia and is known to play an important role in reward learning and action selection [17]. Furthermore, the striatum is related to habit generation when navigating in the environment [18,19]. However, the convergence speed to generate habits is generally long because the state space is continuous. Habits need to be relearned when the environment changes, which shows inflexibility in striatal learning. The prefrontal cortex enables flexible behavior through cognitive control and has the ability to execute a plan in the face of distractions or other forms of interference [20,21]. Prefrontal mediation may be required when both hippocampal and striatal systems are active, and might have a key role in optimizing decision-making strategies [22,23]. Hence, the prefrontal cortex is supposed to receive decisions from the hippocampus and striatum and output the final decision after comprehensive judgment in spatial navigation.

Research in neurophysiology has developed many computing models to explain the navigation mechanism of animals. Stachenfeld et al. proposed the successor representation of the hippocampus, which elaborated that place cells do not encode place per se but rather a predictive representation of future states given the current state [24]. Yu et al. proposed a navigation algorithm for constructing an accurate environmental cognitive map based on the cognitive mechanism of hippocampal space cells [25]. Zhao et al. proposed the prefrontal cortex-basal ganglia algorithm inspired by the mechanism of decision making in the human brain, which uses the actor-critic algorithm to model the dorsal and ventral striatum in the basal ganglia [26]. Most hippocampal and striatal models treat them as independent systems without combining their functions together. However, the interaction between the hippocampus and striatum, also called the hippocampal–striatal circuit, plays an important role in spatial cognition and navigation. McDonald et al. demonstrated incidental hippocampus-based learning when performing a task dependent on the integrity of the dorsolateral striatum, and indicated that the hippocampus obtains information during acquisition of stimulus-response habits [27]. Pezzulo et al. proposed a single mixed instrumental controller, which can produce both goal-directed and habitual behavior based on the hippocampal–striatal circuit [28]. In their model, the goal-directed mechanism is related to model-based reinforcement learning, the habitual mechanism is related to model-free reinforcement learning, and both model-based and model-free mechanisms coexist and compete with each other, the result of which determines operational behavior. However, the mixed instrumental controller does not point out the concrete structural interaction between the hippocampus and striatum in navigation, nor does it involve the formation mechanism of the cognitive map in the hippocampus. In summary, many navigation models focus on one or two parts of the brain area, i.e., the hippocampus, striatum, prefrontal cortex or two of them. Few studies have been conducted to discuss how all of these structures work together in animal navigation and how each of them contributes to the whole navigation learning procedure.

Aiming at the resolution of the problem, we propose a navigation learning model based on the hippocampal–striatal circuit (NLM-HS) that emphasizes the importance of the functional interactions of the hippocampus, striatum and prefrontal cortex, in order to enable agents to navigate similar to animals and to explain in detail how the navigation learning process occurs. The main contributions of the paper can be summarized as follows. Firstly, we combine the three brain structures related to navigation together, i.e., the hippocampus, striatum and prefrontal cortex, to construct a navigation learning model and elaborate their respective roles in navigation. Secondly, the NLM-HS explores how these structures work together to contribute to the whole navigation learning procedure, especially illustrating how goal-directed and habitual navigation strategies switch flexibly during the whole navigation process, which gives a possible explanation for the navigation mechanism in the animal brain. Thirdly, the NLM-HS is used to reproduce the classical Tolman detour experiment to test its effectiveness. Experimental results show that the proposed NLM-HS enables agents to make behavioral decisions faster and to achieve better adaptivity than hippocampal or striatal models alone.

The remainder of this paper is organized as follows. In Section 2, we present the proposed NLM-HS, and introduce the three main components, including the hippocampal model, striatal model and prefrontal cortex model. In Section 3, we show the basic and adaptive navigation results obtained from the NLM-HS, which are compared with those using the hippocampal and striatal models alone in the navigation path, navigation steps and navigation time to test the effectiveness. Then, we develop a discussion in Section 4. Finally, Section 5 concludes the paper.

## 2. Materials and Methods

**Figure 1:**
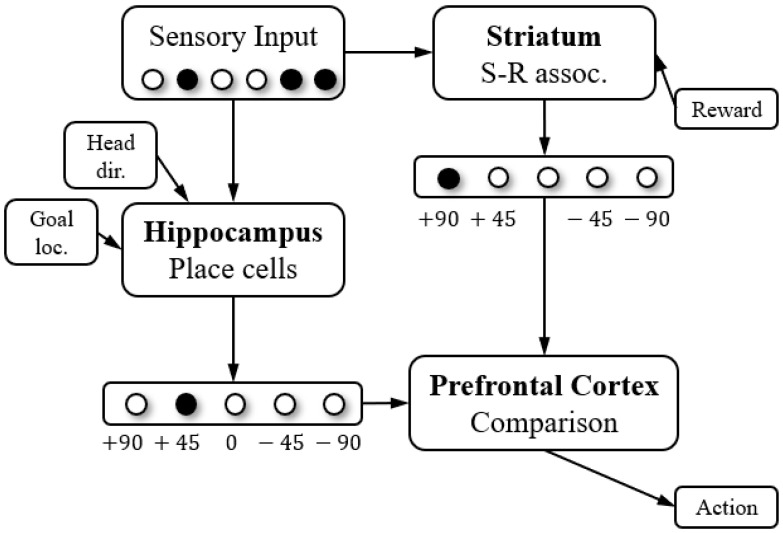
A minimal cognitive architecture for spatial navigation proposed by Chersi et al. [29]. The figure presents a schematic representation of the hippocampal–striatal circuit that guides spatial navigation. The hippocampus and striatum use the same sensory input and output the estimated optimal actions, which are arbitrated and chosen by the prefrontal cortex.

A minimal cognitive architecture for spatial navigation, proposed by Chersi et al. [29] and shown in Figure 1, presents a schematic representation of the hippocampal–striatal circuit that guides spatial navigation. The hippocampus and striatum use the same sensory input and output the estimated optimal actions, which are arbitrated and chosen by the prefrontal cortex. In the architecture, the hippocampus is supposed to provide a cognitive map with information about locations for goal-directed decision making, and the striatum is supposed to learn stimulus-response associations.

### 2.1. Model Construction

Inspired by Chersi’s conceptual cognitive architecture, we established the NLM-HS. To make the model more rigorous, we propose some basic assumptions. Firstly, the agent can recognize surroundings and activate corresponding place cells, which means that each reachable position corresponds to a specific activated place cell, and an activated place cell can express several reachable places within the place field. Secondly, the striatal model uses a cognitive map generated in the hippocampal model as input to learn navigation habits. Thirdly, time proportional to the length of the path is required for the agent to forward sweep in the hippocampus, whereas no time is considered to be consumed when choosing an action using a habit. Fourthly, if the environment changes, the learned habit is considered to be ineffective.

**Figure 2:**
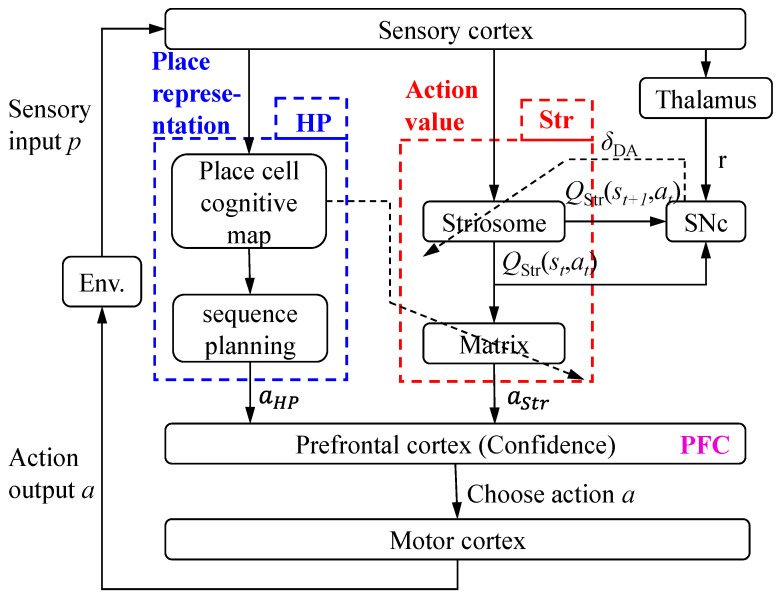
NLM-HS architecture. HP: hippocampus, Str: striatum, PFC: prefrontal cortex, SNc: substantia nigra pars compacta. In the NLM-HS, the functionality of the hippocampal–striatal circuit is designed as follows: The hippocampal model generates a cognitive map of the environment and performs goal-directed navigation. The striatal model performs reward-related habitual navigation. Since the two models may produce inconsistent behavioral decisions, the prefrontal cortex model chooses the most appropriate strategies according to “confidence”. The agent performs the chosen action and gets a new perception, completing an interaction with the environment.

The proposed NLM-HS is composed of the hippocampus, striatum, prefrontal cortex, sensory cortex and motor cortex, as shown in Figure 2. In the NLM-HS, the functionality of the hippocampal–striatal circuit is designed as follows: The hippocampal model generates a cognitive map of the environment and performs goal-directed navigation by using a place cell sequence planning algorithm. The striatal model performs reward-related habitual navigation by using the classic temporal difference learning algorithm. Since the two models may produce inconsistent behavioral decisions, the prefrontal cortex model chooses the most appropriate strategies by using a strategy arbitration mechanism. Meanwhile, the cognitive and learning mechanism of the NLM-HS works in two stages. During the environment exploration stage, the agent uses the hippocampal model to construct the cognitive map of the unknown environment. During the navigation stage, the agent uses the strategy arbitration mechanism in the prefrontal cortex model directly to decide which strategy to choose. When encountering unexpected changes such as obstacles, the agent updates the cognitive map first based on the hippocampal model and then navigates according to the updated cognitive map.

### 2.2. Model of the Hippocampus

The hippocampus is considered to play a central role in spatial representation and episodic memory. In our paper, the hippocampus mainly has two different functions: an environmental cognition function, which can generate and update the cognitive map of the environment, and a decision-making function using a forward sweep process.

**Figure 3:**
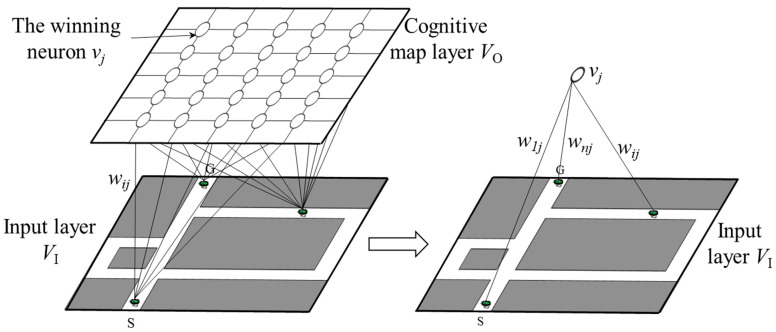
The “sense-response” structure of the DGP-PCCMM. The DGP-PCCMM has two layers: a sensory input layer $V_{I}$ and a cognitive map layer $V_{O}$. As the input of the network, the $V_{I}$ layer interacts with the external environment, sensing and obtaining external information. The $V_{O}$ layer can be seen as the brain, which can form a feature map of the environment. The generated topological map can exist in either of the two forms: the connection weight *W* between the input and output layer or the winning neurons in the output layer. We choose the former to represent the generated cognitive map.

We used the dynamic growing and pruning place cell-based cognitive map model (DGP-PCCMM) [30] to generate a cognitive map of the environment in the hippocampus. The DGP-PCCMM model has two layers: a sensory input layer $V_{I}$ and a cognitive map layer $V_{O}$, as shown in Figure 3. As the input of the network, the $V_{I}$ layer interacts with the external environment, sensing and obtaining external information. The $V_{O}$ layer can be seen as the brain, which can form a feature map of the environment. The generated topological map can exist in either of the two forms: the connection weight *W* between the input and output layer, or the winning neurons in the output layer. We choose the former to represent the generated cognitive map.

The DGP-PCCMM model consists of competition, cooperation and synaptic adaptation stages. In the competition stage, the model obtains the winning neuron through competition:

(1) $$o_{i(x)}(x)=\underset{1\leq j\leq N}{\max}\left\{o_{j}(x)\right\}$$

where x is the sampling input and $o_{i(x)}(x)$ is the output of the winning neuron $v_{i(x)}$. $o_{j}$ is the output of neuron $v_{j}$. $o_{j}=w_{j}^{T}x,j=1,2,\cdots ,N$. To obtain the maximum of $o_{j}$, we can obtain the winning neuron according to

(2) $$i(x)=\arg\underset{1\leq j\leq N}{\min}\left\|x-w_{j}^{T}\right\|$$

In the cooperation stage, as in the biological neural system, the winning neuron activates adjacent neurons with a distribution similar to the Mexican hat function, which can also be a square wave function or a Gaussian function. The most commonly used function is the Gaussian function:

(3) $$\omega_{i(x)j}(t)=\exp\left\{-\frac{d_{i(x)j}^{2}}{2\sigma^{2}}\right\},\;j\in \left\{1,\cdots ,N\right\}$$

where σ is the effective radius of the Gaussian neighborhood and $d_{i(x)j}$ is the Euclidean distance between place cells $w_{j}$ and $w_{i(x)}$.

In the synaptic adaptation stage, the synaptic adaptation law is as follows:

(4) $$\{\begin{array}{c} \Delta w_{j}(t)=\alpha (t)\omega_{i(x)j}(t)\left(x(t)-w_{j}(t)\right)\\ w_{j}(t+1)=w_{j}(t)+\Delta w_{j}(t),j\in \left\{1,\cdots ,N\right\} \end{array}$$

where $w_{j}(t)$ is the feedforward connection strength of neuron j.

The number of neurons in the self-organizing feature map needs to be set in advance, whereas the number of neurons in the DGP-PCCMM model grows dynamically when exploring the environment. The model makes a growth judgment after the competition stage, which is mediated by the growing threshold $V_{\mathrm{GT}}$. If the distance between two place cells $w_{j}$ and $w_{i(x)}$, namely, $d_{i(x)j}$, is larger than the growing threshold $V_{\mathrm{GT}}$, the current mapping network is not considered to be sufficient to map the sampling point, and a place cell and corresponding link between them are added at this time; otherwise, the agent continues to explore the environment.

When exploring an unfamiliar environment, the agent learns to construct a hippocampal cognitive map of the environment, consisting of activated place cells $w_{i(x)}$ and their links. Given enough time, the agent can generate a cognitive map that maps the entire environment. In addition, to strengthen the adaptability of the cognitive map model, we design a dynamic pruning mechanism for the cognitive map. When the agent detects a dynamic obstacle during navigation, the connection relationship between the current place cell and the upcoming place cell is changed to 0, and the distance between the two is considered infinite. Through this mechanism, the connection relationship matrix of the cognitive map can be reduced and updated in real time.

After the exploration stage, the agent uses a place cell sequence planning algorithm to choose actions in the hippocampus, which can be regarded as a forward sweep process in the hippocampus. Inspired by the goal orientation of animal navigation, the negative orientation function is defined as the energy consumption from the current place cell to the goal place cell. The longer the path is, the more energy consumption, and the greater the value of the negative orientation function. The agent chooses the adjacent place cell whose negative orientation function is the smallest among all possible place cells and adds it to the navigation path; a process that is iterated until the agent reaches the goal place cell and completes one navigation episode. The decision-making time using a forward sweep is relatively long.

### 2.3. Model of the Striatum

The striatum receives sensory information from the sensory cortex and activated place cells from the hippocampus. We use the classical temporal difference (TD) learning algorithm to learn the generation of habitual navigation in the striatum. The striatum consists of the striosome and the matrix. The striosome outputs behavioral evaluation information, and the matrix performs action selection according to the behavioral evaluation information and improved ε-greedy algorithm. The framework of the striatal model is shown in the red section in Figure 2.

The agent receives an immediate reward at time t, which is represented by $r_{t}$. When the agent activates a place cell, the striosome outputs the behavioral evaluation information, i.e., expected discounted return at time t:

(5) $$Q_{\mathrm{Str}}(s_{t},a_{t})=r_{t+1}+\gamma r_{t+2}+\gamma^{2}r_{t+3}+\cdots$$

where γ is the discount rate, and 0≤γ≤1. The expected discounted return at time t+1 is:

(6) $$Q_{\mathrm{Str}}(s_{t+1},a_{t+1})=r_{t+2}+\gamma r_{t+3}+\gamma^{2}r_{t+4}+\cdots$$

From Formulas (5) and (6), we know:

(7) $$Q_{\mathrm{Str}}(s_{t},a_{t})=r_{t+1}+\gamma Q_{\mathrm{Str}}(s_{t+1},a_{t+1})$$

which shows that the expected discounted return at time t, $Q_{\mathrm{Str}}(s_{t},a_{t})$, can be represented by the expected discounted return at time t+1, $Q_{\mathrm{Str}}(s_{t+1},a_{t+1})$. However, as there will be an error in the early prediction, $Q_{\mathrm{Str}}(s_{t},a_{t})$ expressed by $Q_{\mathrm{Str}}(s_{t+1},a_{t+1})$ is not equal to the actual $Q_{\mathrm{Str}}(s_{t},a_{t})$. Thus, the reward information from the striosome and thalamus is processed in the substantia nigra pars compacta (SNc), producing a dopamine response:

(8) $$\delta_{\mathrm{DA}}=r_{t+1}+\gamma Q_{\mathrm{Str}}(s_{t+1},a_{t+1})-Q_{\mathrm{Str}}(s_{t},a_{t})$$

The agent updates the reward information $Q_{\mathrm{Str}}(s_{t},a_{t})$ every time it passes through a place cell:

(9) $$Q_{\mathrm{Str}}(s_{t},a_{t})=Q_{\mathrm{Str}}(s_{t},a_{t})+\alpha \cdot \delta_{\mathrm{DA}}$$

where α is the learning rate and 0≤α≤1. According to the action evaluation $Q_{\mathrm{Str}}(s_{t},a_{t})$ from the striosome, the matrix in the striatum chooses an action using an improved ε-greedy algorithm:

(10) $$\pi \left(s_{t}\right)=\{\begin{array}{c} \begin{array}{cc} \mathrm{random}\;\mathrm{action}\;a\in \Omega , & if\;\xi <\varepsilon \end{array}\\ \begin{array}{cc} \underset{a\in \Omega }{\arg\max Q_{\mathrm{Str}}\left(s_{t},a_{t}\right),} & otherwise \end{array} \end{array}$$

In contrast to the traditional ε-greedy algorithm with a fixed ε value, the improved ε-greedy algorithm sets the exploration rate to decrease over time:

(11) ε = κ1⋅e−(κ2tN)

where $\kappa_{1}$ and $\kappa_{2}$ are the exploration rate coefficients, t is the navigation episode, and N is the total number of navigation episodes.

The striatal model learns the egocentric stimulus-response connection. The agent receives an immediate reward at the goal, causing the SNc to release a dopamine signal that acts as an incentive to navigate to the goal. After many navigation episodes, the agent can obtain the expected discounted return, not the instant reward distribution, of different place cells in the maze.

Behavioral shaping involves reinforcing the behavior by pairing a behavior with reward in a stepwise manner that is successively closer to a desired behavior [31]. The functional impact of habituation is thought to minimize redundant information, filter input and enhance the novelty of stimuli. Because of the egocentric nature of sensory input, distal directional cues are not particularly prominent. Thus, the striatum will learn to associate egocentric sensory representations with egocentric behaviors that lead to a reward.

In episodic tasks, we need to distinguish the set of all nonterminal states, which is a critical problem in robot navigation tasks for naturally unidentifiable states. The most useful method is to divide the environment as a grid map, and each grid represents a state. The size of the grid map affects the accuracy of navigation. The artificial division of the grids makes the robot lack intelligence. However, the hippocampal cognitive map is an effective representation of the environment, which can be used as the input of the striatum to provide navigation states for navigation.

### 2.4. Model of the Prefrontal Cortex

Given the potentially different action outputs from the hippocampal and striatal models, it is necessary to build an arbitration mechanism and decide the appropriate action. A potential component arbitrating these two systems is the prefrontal cortex. Goal-directed decision making takes place mainly in the hippocampus, which is flexible but slow, whereas habitual decision making is performed primarily by the striatum, which is inflexible but fast. The significance of the arbitration mechanism in the prefrontal cortex is that it takes advantage of the two models to enable the agent to complete navigation efficiently.

The prefrontal model chooses action primarily according to the value of “confidence”, which is defined by its influencing factors. In different navigation stages, the “confidence” values of different strategies are different. We define the “confidence” of strategies as:

(12) $$\mathrm{CONF}=\frac{1}{U\cdot R}$$

where U is the “uncertainty” of the model and R reflects the “rapidity”, which is calculated by the time consumed to make decisions during the navigation process. The more time consumed, the larger R is. The agent chooses a strategy with high “confidence” to navigate effectively.

Due to the existence of cognitive maps, the uncertainty of decision making in the hippocampal model can be regarded as a fixed value, which is roughly the same as the uncertainty of the striatal model after habituation. The uncertainty of habitual navigation strategies in striatal models is related to the number of navigations and whether there are changes in the environment. Thus, we define the “uncertainty” of the striatal model as:

(13) $$U_{\mathrm{Str}}=\{\begin{array}{cc} +\infty & N_{\mathrm{epi}}<N_{\mathrm{habit}}\\ 1 & N_{\mathrm{epi}}>N_{\mathrm{habit}}\;\&\;c=0\\ +\infty & N_{\mathrm{epi}}>N_{\mathrm{habit}}\;\&\;c=1 \end{array}$$

where $N_{\mathrm{epi}}$ is the navigation episode, $N_{\mathrm{habit}}$ is the navigation episodes required to generate a habit in the environment, c=0 indicates that the environment has not changed, and c=1 indicates that the environment has changed. Then, since the forward sweep takes time, the hippocampal model takes a longer time than the habitual navigation of the striatum. Therefore, the relationship of rapidity R between the two systems is $R_{\mathrm{HP}}>R_{\mathrm{Str}}$.

The “confidence” in the prefrontal cortex can be represented by:

(14) $${\mathrm{CONF}}_{\mathrm{PFC}}={\mathrm{CONF}}_{\mathrm{HP}}-{\mathrm{CONF}}_{\mathrm{Str}}$$

in which ${\mathrm{CONF}}_{\mathrm{HP}}$ and ${\mathrm{CONF}}_{\mathrm{Str}}$ are confidence values of strategies in the hippocampus and striatum, respectively, which are computed by Formula (12). If ${\mathrm{CONF}}_{\mathrm{PFC}}>0$, which indicates that the confidence in the hippocampus is higher than the confidence in the striatum, the agent chooses actions from the hippocampus. Conversely, if ${\mathrm{CONF}}_{\mathrm{PFC}}<0$, the agent chooses actions from the striatum. A qualitative analysis of “confidence” in the prefrontal cortex is applied based on the experimental process, which can be classified into 4 periods, as shown in Table 1.

As Table 1 shows, the agent chooses action in the prefrontal cortex according to whether the behavioral habit is formed. When the agent is placed in an unknown environment, the agent initially explores the environment randomly and forms a cognitive map according to the hippocampal model. Given enough time, the agent can traverse the environment sufficiently and form a cognitive map that maps the whole environment. Then, the agent begins the navigation process. When the agent performs actions, the hippocampal and striatal models make behavioral decisions simultaneously, and the prefrontal cortex model selects one of them to execute the decision based on the “confidence”. Although only one of the decision systems is chosen at one navigation episode, the other system also performs learning at the same time. For example, at the initial time, the agent uses a strategy in the hippocampus to navigate, but the striatal model also learns the stimulus-response associations, represented by behavioral evaluation information $Q_{\mathrm{Str}}(s_{t},a_{t})$. More training episodes result in more optimized behavioral evaluation information $Q_{\mathrm{Str}}(s_{t},a_{t})$. Given enough time, the agent forms behavioral habits leading gradually to habitual selection, which is expected to accelerate the convergence speed of navigation. When the environment changes, the agent uses the hippocampal model to update the cognitive map. At the same time, the agent needs to relearn the striatal model and reconstruct a new behavioral habit. The integrated navigation algorithm is shown in Table 2.

**Figure 4:**
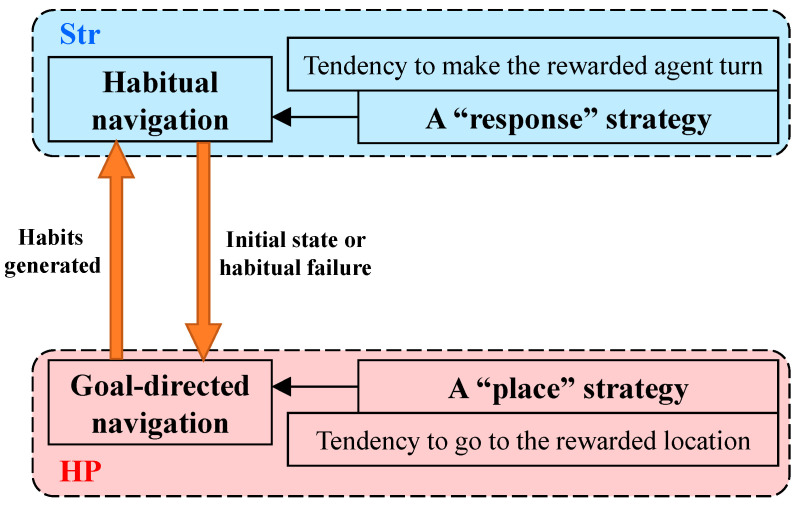
Relationship between habitual and goal-directed navigation. The agent chooses a path according to the goal-directed mechanism until it has acquired enough experience and formed habits and then navigates according to habits, which is consistent with animal navigation behavior.

When spatial knowledge is used to plan a route to the goal, the agent either follows a familiar route or calculates a new route based on the cognitive map. That is, the navigation strategy of the agent switches flexibly between habitual navigation and goal-directed navigation, as shown in Figure 4. Since behavioral habits have not been formed in the initial stage of navigation, agents tend to use goal-directed strategies in hippocampal models. Later, the agent tends to choose a habitual strategy when behavioral habits are formed. That is, the agent chooses a path according to the goal-directed mechanism until it has acquired enough experience and formed habits, and then navigates according to habits, which is consistent with animal navigation behavior. In goal-directed tasks, rats are able to learn reward locations in the environment and unlearn them when they are changed. When the reward is removed or the environment changes, the agent can learn the change and gradually form new behavioral habits.

The main advantage of the NLM-HS lies in the arbitration mechanism in the prefrontal cortex between the hippocampal and striatal systems. By comparing the “confidence” of the two systems, the agent can choose the most appropriate actions at each activated place cell. The hippocampal model makes mainly goal-directed decisions based on the overall cognitive map so that the agent tends to go to the rewarded location in the environment, whereas the striatal model focuses on making the rewarded agent turn and eventually leads to habitual selection. Complementing hippocampal spatial coding, the striatal model provides an action value and reward for decision making. The NLM-HS is expected to obtain the optimal choice between the two navigation strategies.

## 3. Results

To verify the correctness and effectiveness of the NLM-HS, the basic and adaptive navigation experiments were carried out with the hippocampal model alone, the striatal model alone and the NLM-HS model, respectively. We analyze the performance of each model through navigation trajectory, navigation steps, and navigation time. Here, “navigation steps” refers to the number of running steps. The result section is developed as follows. First, experiment design is introduced in Section 3.1. Then, the basic and adaptive navigation experiments are illustrated in Section 3.2 and Section 3.3, respectively. Finally, we set out the results comparison and analysis in Section 3.4. It should be pointed out here that we have carried out several navigation experiments on the three models. When we show the results, we first show the results of one round navigation experiment of the three models, and then give the comparison results of the statistical values of many experiments in Section 3.4.

### 3.1. Experiment Design

**Figure 5:**
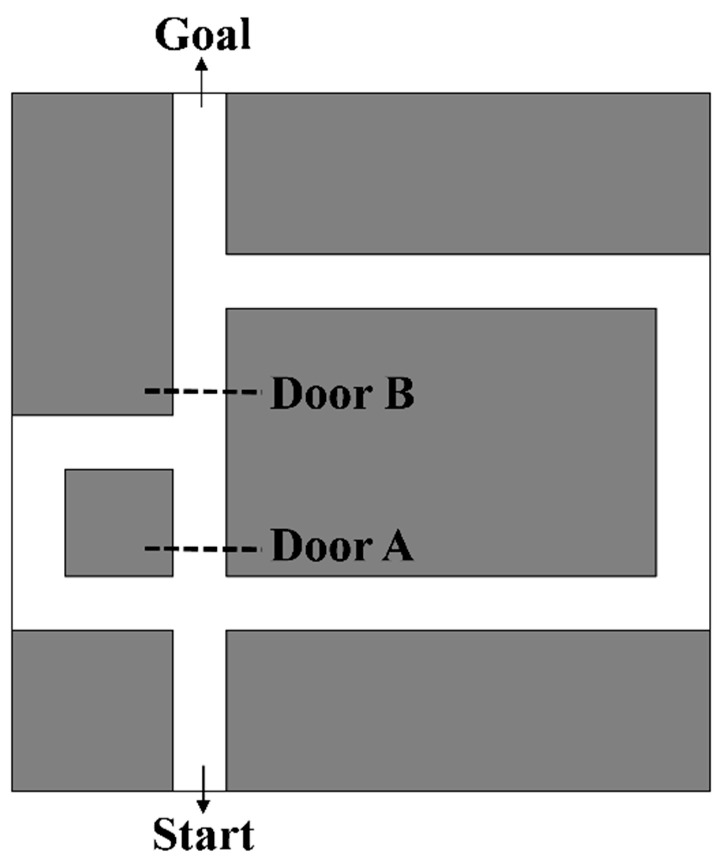
Tolman detour maze environment. The maze consists of three passages, and doors A and B are set in the passage to provide the dynamic environment. The size of the maze is set to 130 cm × 130 cm, and the coordinates of the start and goal are (35, 5) and (35, 125), respectively.

One way to verify whether an agent owns navigation intelligence is to have the agent perform tasks similar to animal navigation and compare the results. In this paper, we conducted a series of simulation experiments based on the Tolman detour maze environment, which consists of three passages, and doors A and B are set in the passage to provide the dynamic environment [32], as shown in Figure 5. The size of the maze is set to 130 cm × 130 cm, and the coordinates of the start and goal are (35, 5) and (35, 125), respectively.

Inputs of the hippocampal and striatal models are positions of the agent. The agent can obtain current location information by sensing the surroundings, which belongs to the research category of robot localization and will not be elaborated on specifically here. During the environmental cognition stage, the agent explores the environment randomly and current positions of the agent are used as input of our models.

In the hippocampal model, the output shows different functions according to different stages. During the environmental cognition stage, the hippocampal model commits to forming a cognitive map of the environment, which is represented by the activated place cells and the links among them. During the navigation stage, the hippocampal model commits to choosing actions according to the place cell sequence planning algorithm. There are eight kinds of actions at one activated place cell, including moving along each of the cardinal and intercardinal directions.

In the striatal model, the output is an action chosen by behavioral habits. In the early navigation stages, the striatal model did not generate behavioral habits, so the actions are chosen randomly. After a period of navigation learning, the agent gradually forms behavioral habits, which enable agents to reach the goal faster.

In the proposed NLM-HS model, the agent performs an action only during one step, so it is very likely that a randomly selected action in the striatal model will not be selected in the early navigation stages. Instead, the action chosen by the hippocampal model will be selected.

### 3.2. Basic Navigation Experiments

In the basic navigation experiments, where door A and door B were both open, the agent performed navigation with the hippocampal model alone, the striatal model alone and the NLM-HS model.

#### 3.2.1. Navigation with the Hippocampal Model Alone

**Figure 6:**
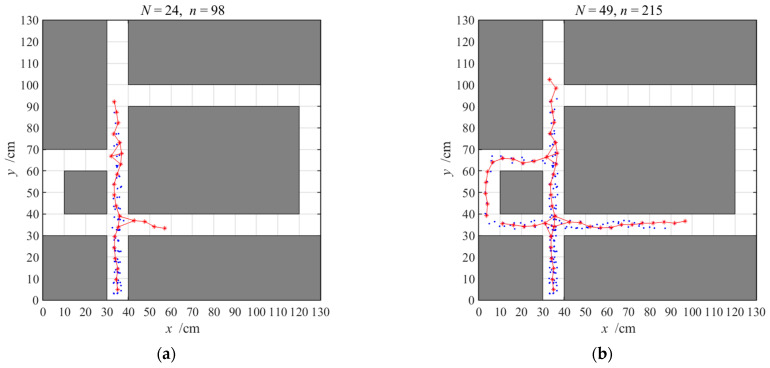

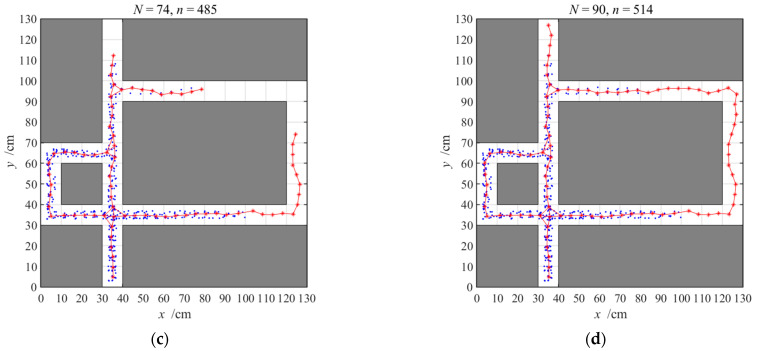
The generation process of the cognitive map; (**a**–**d**) are the cognitive maps when 24, 49, 74, and 90 place cells are activated, respectively. The reachable positions, the activated place cells and the links between them are marked by blue points, red asterisks and red lines, respectively. *N* at the top of each panel represents the number of activated place cells, and *n* represents the number of positions passed.

As mentioned above, the hippocampal model has the function of environmental cognition and decision making for goal-directed navigation. First, the agent generates the cognitive map of the environment using the hippocampal model when it is in an unfamiliar environment. The agent can obtain the activated place cells and the links among them by interacting with the environment. In the DGP-PCCMM, the output numbers need not be set in advance; in contrast, the output numbers grow dynamically with the navigation process. We set the growing threshold $V_{\mathrm{GT}}$ to 4.5, set the exploring steps to 1000 and record the cognitive map every time 25 place cells are activated. Then, we can obtain the generation process of the cognitive map, as shown in Figure 6, from which we can see that environmental cognition is a gradual process and the entire maze can be represented by only 90 activated place cells. We can conclude that the agent can express the whole infinite environment with finite activated place cells, which can be regarded as a possible way to understand the environment in the animal brain.

**Figure 7:**
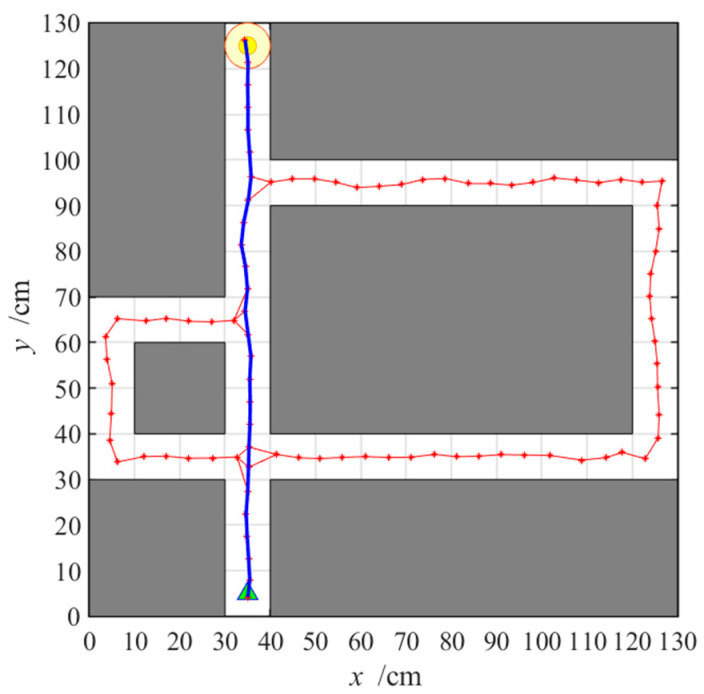
Navigation path with the hippocampal model alone. Red asterisks and the red lines linking them mark the generated cognitive map. Blue lines represent navigation path.

**Figure 8:**
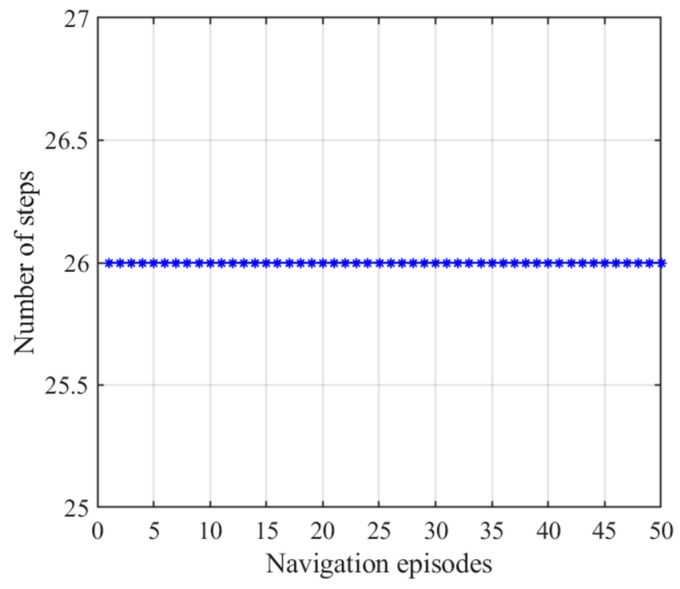
Number of steps with the hippocampal model alone. Blue asterisks indicate the navigation steps for each navigation episode.

**Figure 9:**
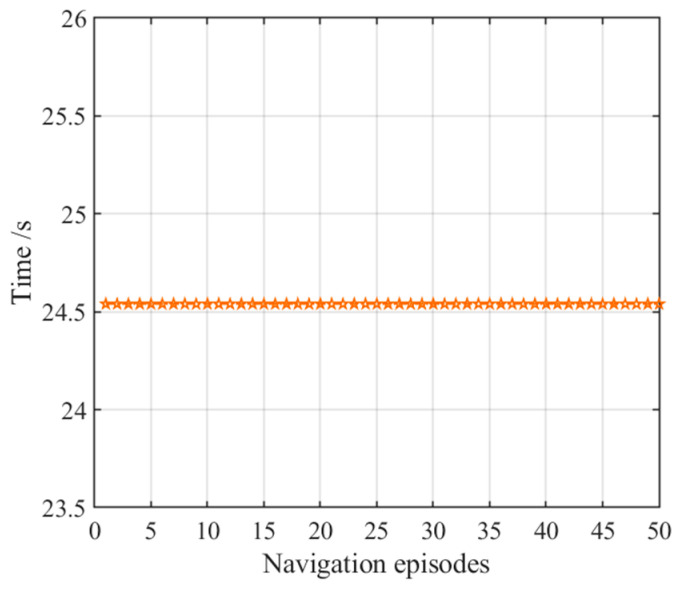
Navigation time with the hippocampal model alone. Orange pentagrams indicate the navigation time for each navigation episode.

After obtaining the cognitive map of the environment, the agent begins the navigation stage, which is achieved by the place cell sequence planning algorithm in the hippocampal model. The navigation path of the hippocampal model is shown in Figure 7, and the number of running steps and navigation time of the 50 navigation episodes are shown in Figure 8 and Figure 9, respectively, from which we can see that the navigation steps and navigation time do not change with navigation episodes, resulting from the fact that the knowledge base, namely, the cognitive map, formed in the hippocampal model is basically fixed as long as the agent is in a static environment. The agent chooses actions at each place cell according to the place cell sequence planning algorithm, which can be regarded as a forward sweep process. Navigation with the hippocampal model alone needs to consume a certain decision time, which can be calculated from the ratio of the navigation path to the step length, as shown in Figure 9.

#### 3.2.2. Navigation with the Striatal Model Alone

When navigating with the striatal model alone, we mark the boundary of place cells every 5 cm from zero in the positive directions of the x- and y-axes to. In striatal TD reinforcement learning, we set the discount rate γ to 0.9 and the learning rate α to 0.9. The agent receives a penalty of −0.1 for every further step forward and a reward of 300 for reaching the goal position. We set the exploration rate to decrease over time:

(15) ε = 0.6×e−(tN)

**Figure 10:**
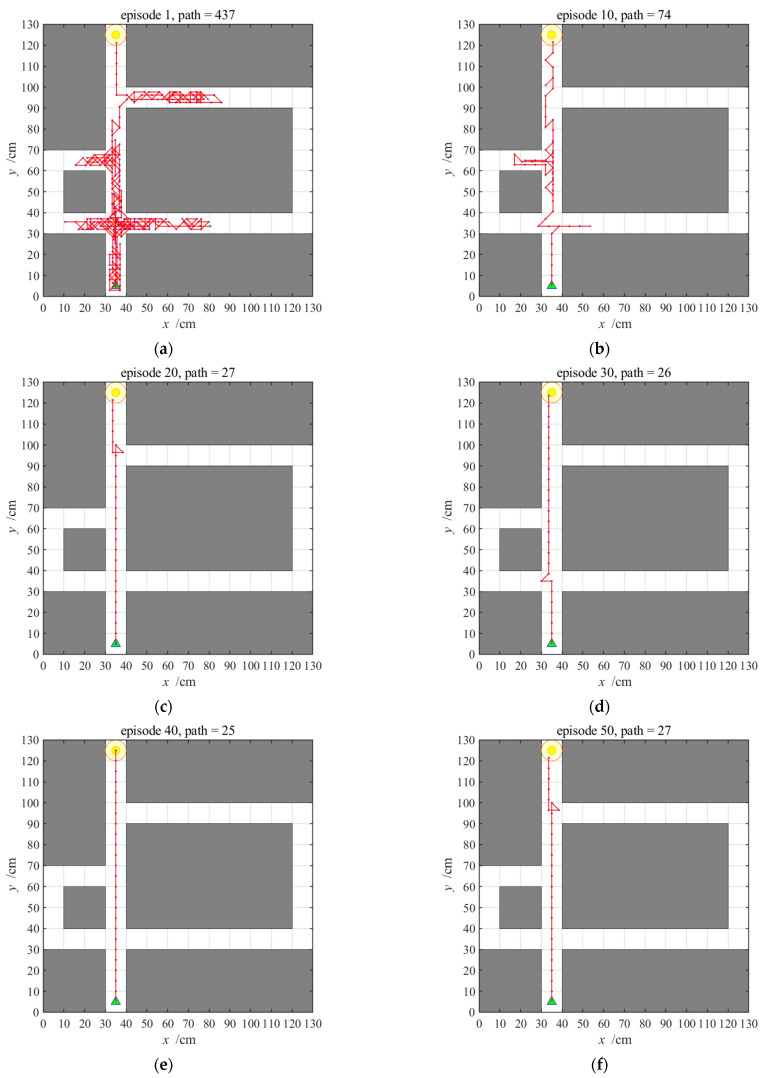
Navigation path with the striatal model alone; (**a**–**f**) show navigation paths in the 1st, 10th, 20th, 30th, 40th, and 50th episodes, respectively. The episode number and the path length measured by the steps in each experiment are shown at the top of the panels.

The navigation path with the striatal model alone is shown in Figure 10. We select the 1st, 10th, 20th, 30th, 40th, and 50th navigation episodes to display. From the six paths, we can see that agents will take long detours during earlier navigation processes, which constitute a kind of trial-and-error navigation. After a certain amount of navigation, the path gradually tends to be optimal.

**Figure 11:**
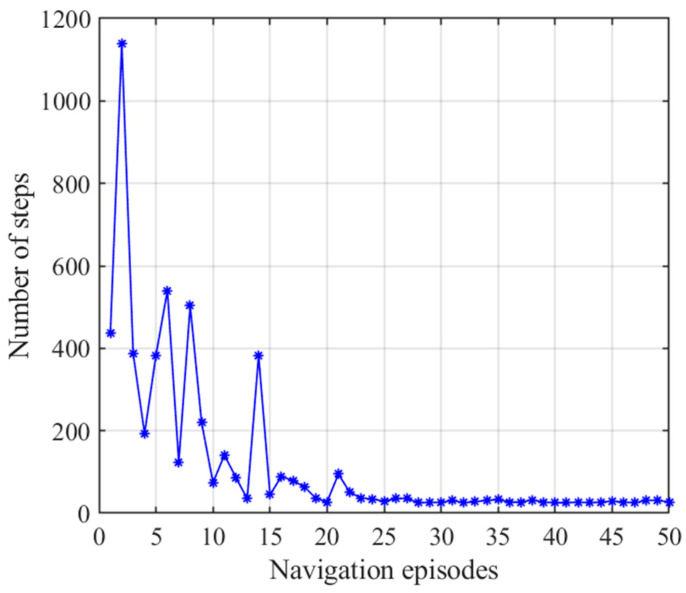
Number of steps with the striatal model alone. Blue asterisks indicate the navigation steps for each navigation episode.

**Figure 12:**
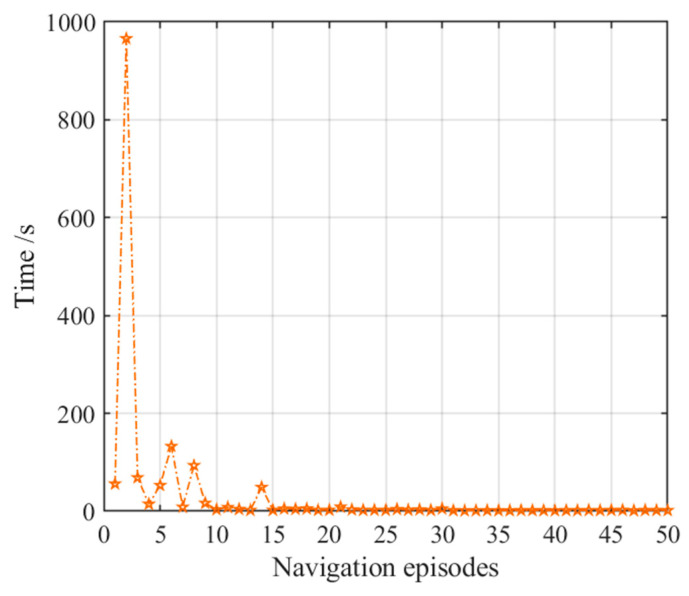
Navigation time with the striatal model alone. Orange pentagrams indicate the navigation time for each navigation episode.

The number of steps and navigation time in the 50 navigation episodes with the striatal model alone are shown in Figure 11 and Figure 12, respectively, which show that the number of steps and navigation time used to reach the goal are very large during the earlier few navigation episodes and decrease gradually until they become stable. This is because the agent is initially unfamiliar with the environment and it requires a period of time to interact with and learn from the environment. The ability to converge gradually to an optimal navigation path is closely related to the characteristics of reinforcement learning of the striatal model. The knowledge base of the agent is a cumulative process from having nothing to having knowledge, and from less to more, and the agent eventually learns to select the optimal strategy. Although the agent can obtain a relatively optimal path and form a behavioral habit of navigation, Figure 11 and Figure 12 also tell us that the convergence speed of navigation based on the striatal model alone is very slow and that the habit-generation process consumes considerable time, which shows that implementing the striatal model alone cannot realize effective navigation.

#### 3.2.3. Navigation with the NLM-HS

**Figure 13:**
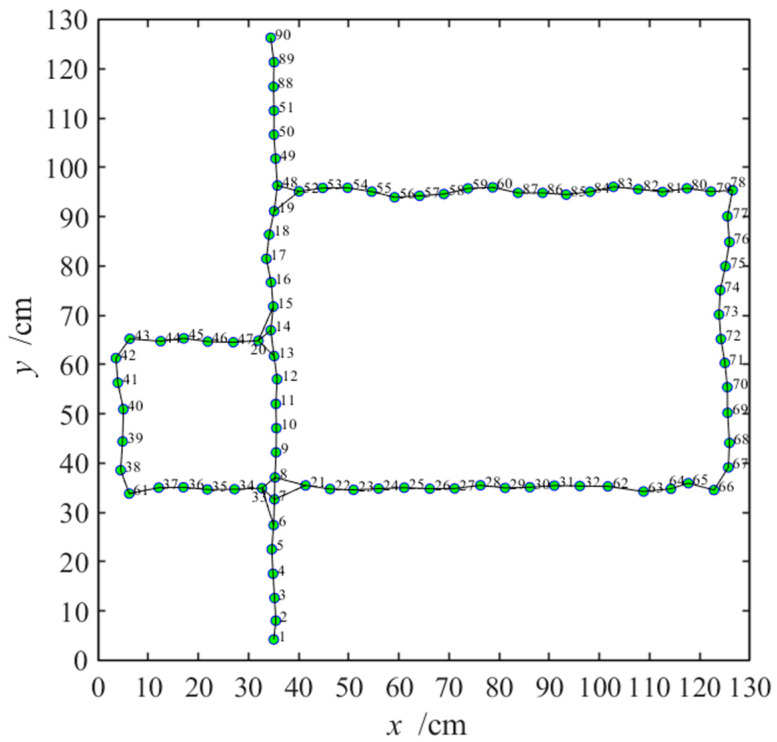
Cognitive map with numbered place cells according to the generation sequence. The green circles and black lines represent the cognitive map. Numbers next to the circle represent the generation order of place cells.

When navigating with the NLM-HS, the agent first explores the environment to obtain a hippocampal cognitive map and then uses the arbitration mechanism of the prefrontal cortex model to choose a behavioral strategy. The generation process for the cognitive map is shown in Figure 6. At the same time, we numbered the place cells according to the generation sequence, and we can obtain the cognitive map with numbered place cells, as shown in Figure 13, which can be used as a discretized state when learning using the striatal model.

In this paper, we set out the navigation episodes required to generate habit $N_{\mathrm{habit}}$ as a constant. We believe that in the specific environment, the minimum number of navigation episodes required to generate habits is 30. When the environment changes or a detour is required, the minimum number of navigation episodes required to generate habit is 1.2 times the number of navigation steps at the 30th navigation episode.

**Figure 14:**
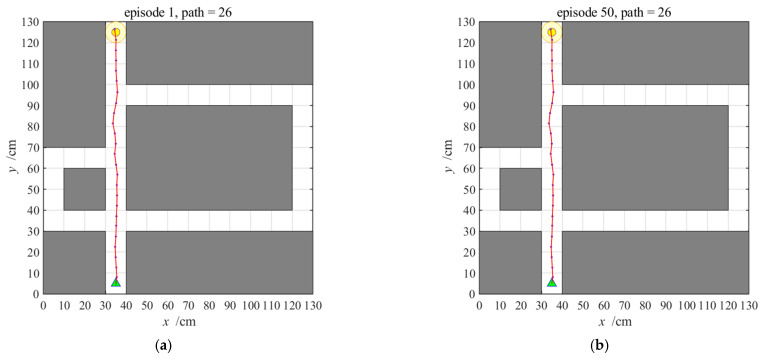
Navigation path with the NLM-HS; (**a**,**b**) represent the 1st and 50th navigation paths, respectively.

Figure 14 provides the navigation path of the NLM-HS. Initially, the agent uses the place cell sequence planning mechanism in the hippocampal model for navigation. At the same time, the striatal model also learns the stimulus-response association in the environment. After a period of learning, the striatal model learned the behavioral habit, according to which the agent can achieve rapid navigation. Figure 14 shows that the navigation path is the same using both hippocampal and striatal models, due mainly to the use of the cognitive map generated during the exploration stage for the two models.

**Figure 15:**
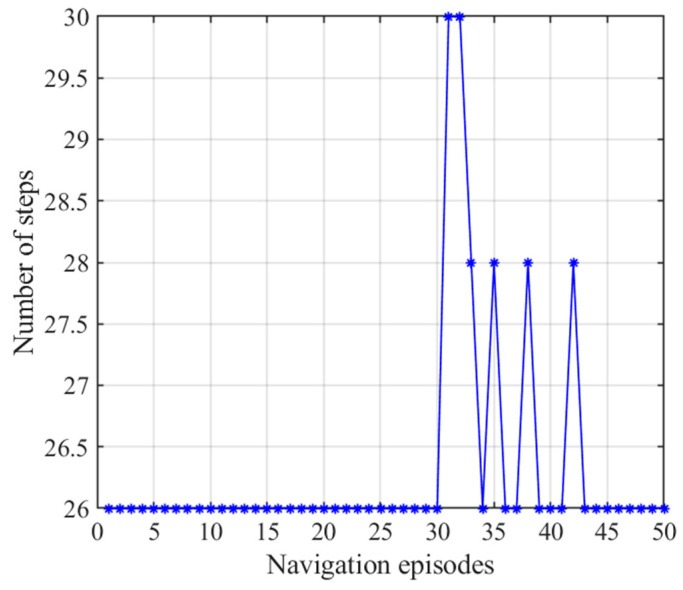
Number of steps with the NLM-HS. Blue asterisks indicate the navigation steps for each navigation episode.

**Figure 16:**
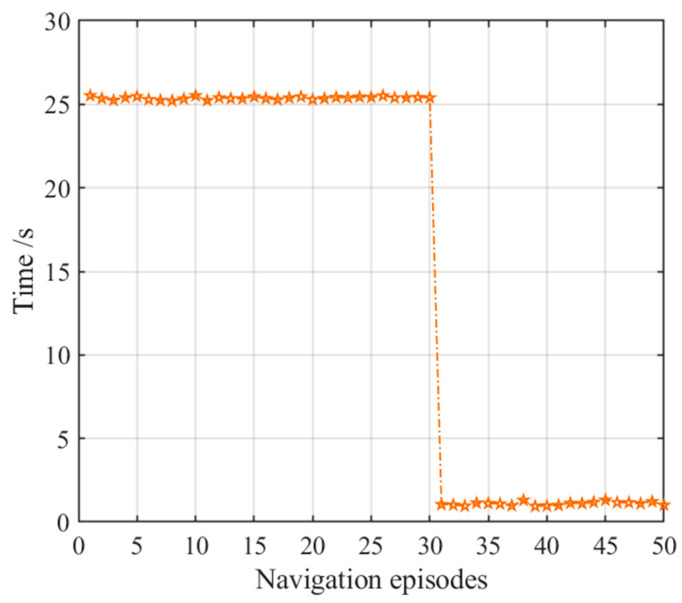
Navigation time with the NLM-HS. Orange pentagrams indicate the navigation time for each navigation episode.

Figure 15 presents the number of steps of the NLM-HS during 50 navigation episodes, showing that the number of navigation steps needed to reach the goal remains basically the same in the earlier 30 navigation steps, and then stabilized again after a slight fluctuation after the 30th navigation episode. This is because the agent starts to adopt the strategy generated from the striatal model after the 30th navigation episodes, and the striatal model has a certain exploration rate. Although there are small fluctuations in the path, the navigation time is greatly reduced, as shown in Figure 16. Figure 16 shows the variation in navigation time with the NLM-HS, which presents a sudden drop at the 30th navigation episode. Compared with the hippocampal model alone, the time used with the NLM-HS is less. Compared with the striatal model alone, the NLM-HS is more efficient because it does not need redundant learning to reach the goal. The NLM-HS is rooted in the fact that it can combine the two models’ respective advantages, namely that the hippocampal model can generate, update and perfect the knowledge base through learning, while the striatal model can accelerate the navigation process by using behavioral habits.

### 3.3. Adaptive Navigation Experiments

To explore the adaptive abilities of the NLM-HS, we studied the navigation changes of the three models when encountering a sudden change in the environment. In this paper, door B closed.

**Figure 17:**
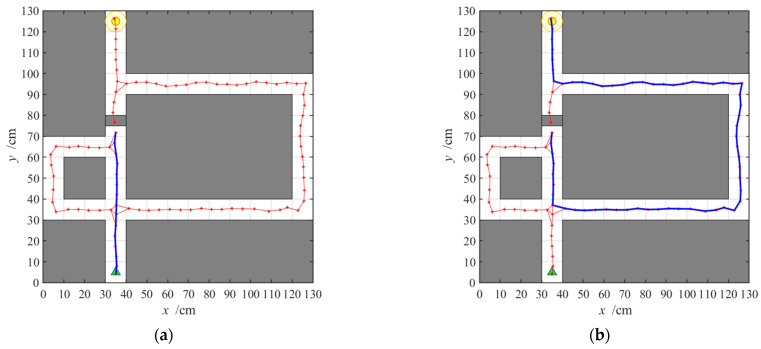

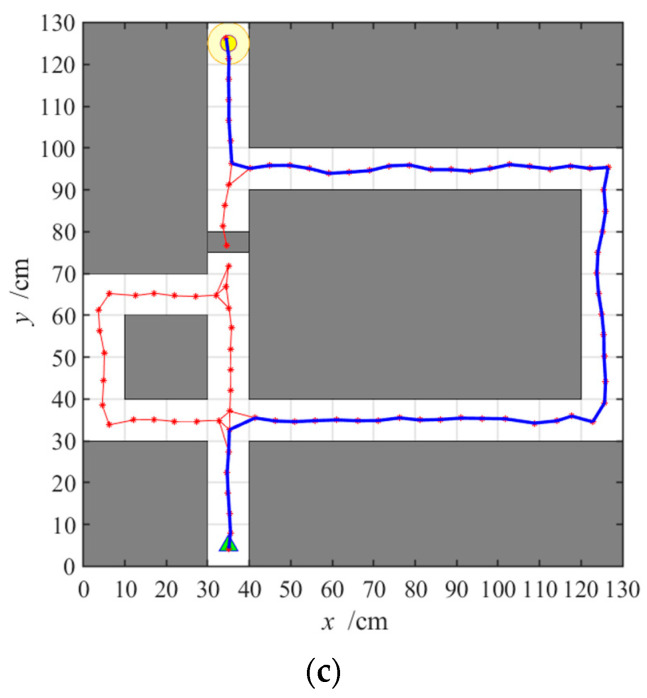
Navigation path with the hippocampal model alone when door B is closed; (**a**) shows the agent’s encounter with the closed door B, and (**b**) shows the newly planned navigation path from current position, both of which constitute the first navigation path. In addition, (**c**) shows navigation path after first navigation.

When the hippocampal model alone is used for navigation, the agent will update the cognitive map when encountering door B by using a dynamic pruning algorithm, and then use the place cell sequence planning algorithm for real-time path planning. The first path and the eventually converged navigation path are shown in Figure 17. When the agent encounters door B, it cuts off the link first, namely, it sets the connection between the current place cell and the upcoming place cell to 0. Then, the agent starts from the currently activated place cell and reuses the place cell sequence planning algorithm to plan the path. The first navigation when door B is closed consists of the two periods shown in Figure 17a,b, and we can obtain the number of navigation paths as 76 and the navigation time as 74.99 s. Starting from the second navigation, the agent directly navigates according to the path obtained by the forward sweep and can obtain the number of navigation paths as 61 and navigation time as 60.30 s.

**Figure 18:**
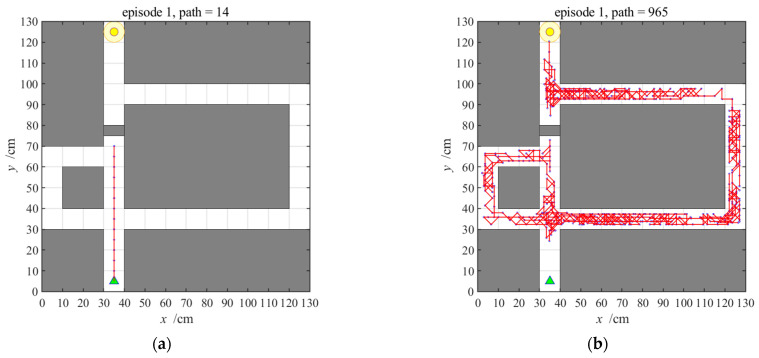

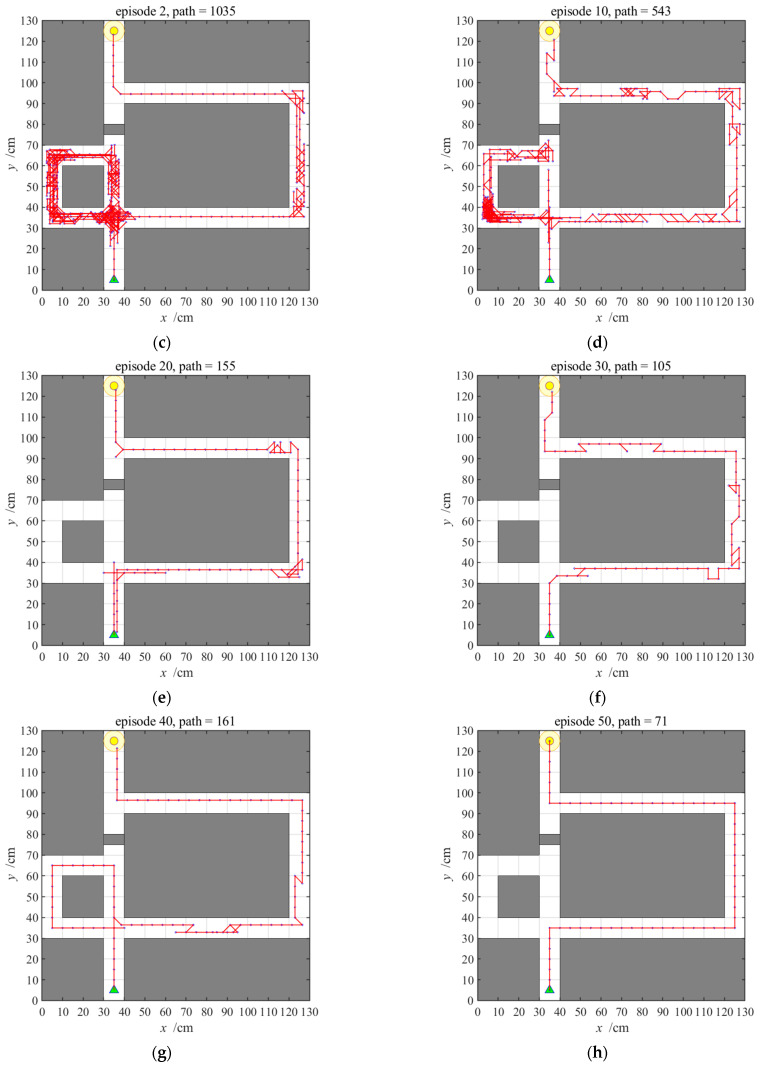
Navigation path with the striatal model alone when door B is closed; (**a**) shows the agent’s encounter with the closed door B, and (**b**) shows the new navigation path from current position, both of which constitute the first navigation path. In addition, (**c**–**h**) show navigation paths in the 2nd, 10th, 20th, 30th, 40th, and 50th episodes, respectively. The episode number and the path length measured in steps for each experiment are shown at the top of the panels.

**Figure 19:**
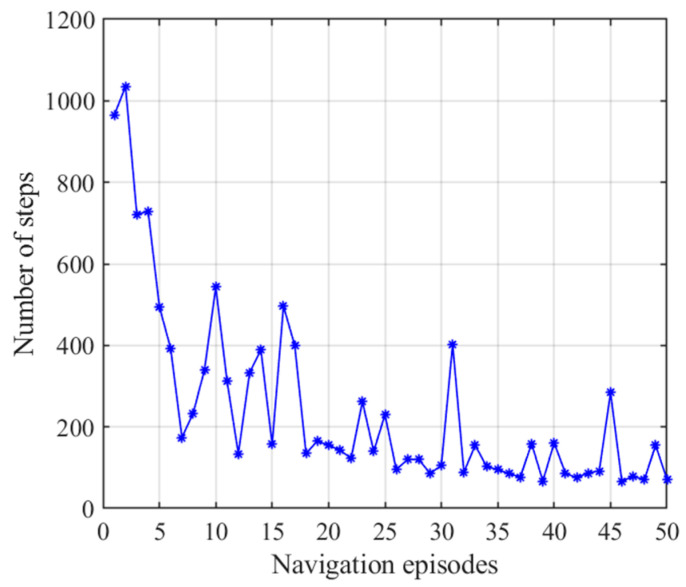
Number of steps with the striatal model alone when door B is closed. Blue asterisks indicate the navigation steps for each navigation episode.

**Figure 20:**
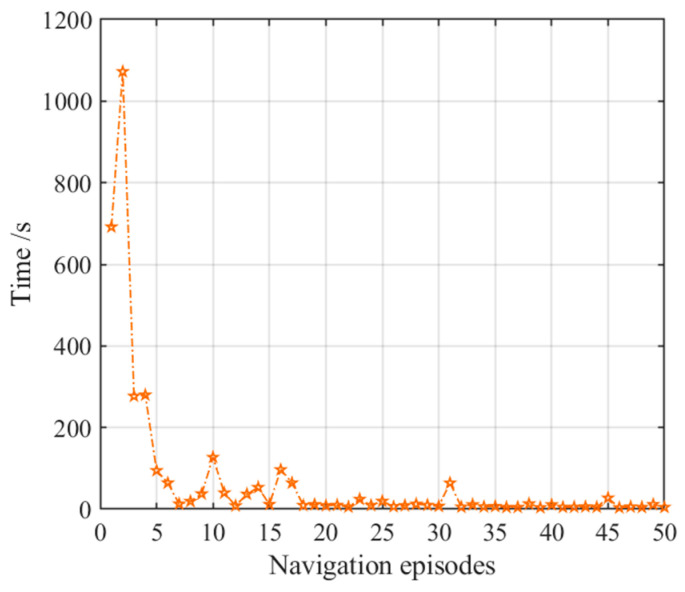
Navigation time with the striatal model alone when door B is closed. Orange pentagrams indicate the navigation time for each navigation episode.

When the striatal model alone is used for navigation, the agent navigates according to a habitual strategy. When the agent encounters closed door B, the previously generated habit becomes invalid. It is necessary to re-explore the environment and generate a new habit. The navigation process is shown in Figure 18, and the number of steps and navigation time changes are shown in Figure 19 and Figure 20, respectively. The agent initially uses the learned habit to navigate until it encounters closed door B, and then the agent starts from the current position and re-explores the environment to learn new habits. The first navigation path when door B is closed consists of the two periods, shown in Figure 18a,b. By constant navigation learning, the agent learns a new navigation habit. From Figure 19, we can see a relatively large fluctuation, while the number of navigation steps declines, which shows that using the striatal model alone has poor adaptability, thus failing to achieve a better navigation effect.

**Figure 21:**
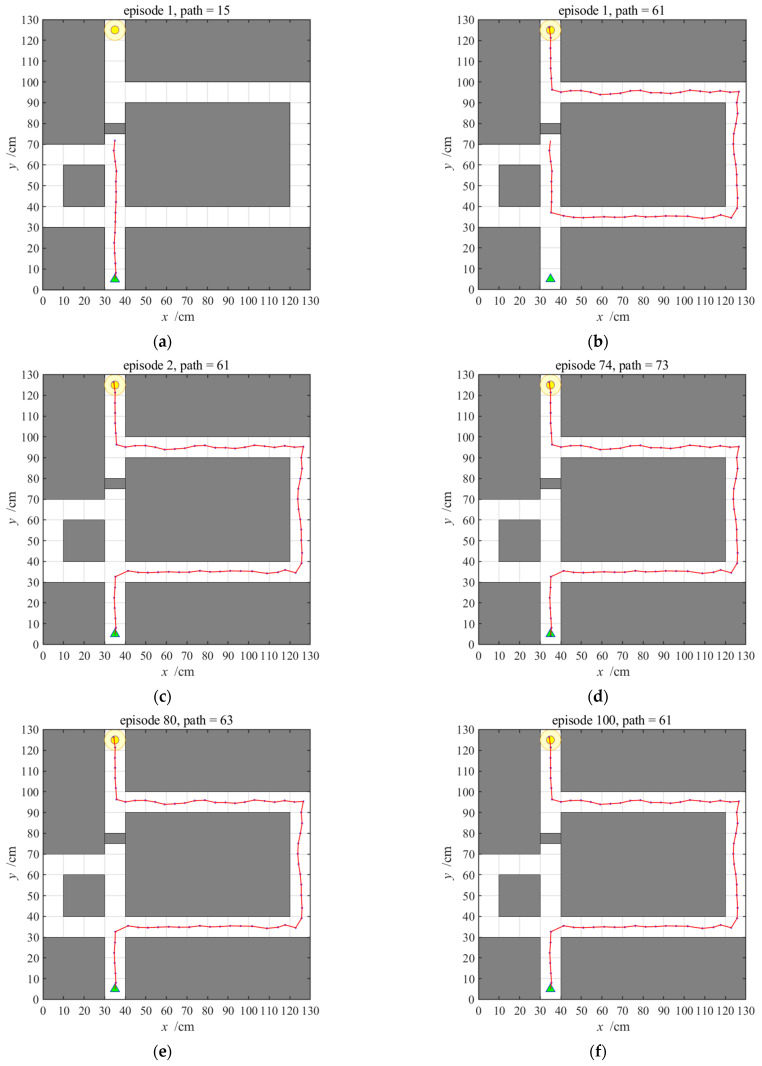
Navigation path with the NLM-HS when door B is closed; (**a**) shows the agent’s encounter with the closed door B, and (**b**) shows the new navigation path from current position, both of which constitute the first navigation path. In addition, (**c**–**f**) show navigation paths in the 2nd, 74th, 80th and 100th episodes, respectively. The episode number and the path length measured by the steps in each experiment are shown at the top of the panels.

When the NLM-HS is used for navigation, the agent first navigates according to a habitual strategy and then needs to re-explore the environment and generate new habits when encountering closed door B. Since the uncertainty of the striatal model increases, the prefrontal cortex starts choosing the behavioral strategy in the hippocampal model. The agent updates the cognitive map using a dynamic pruning algorithm and performs actions according to the place cell sequence planning algorithm in the hippocampal model. At the same time, the striatal model also learns the stimulus-response associations by interacting with a new environment and gradually obtains new habits because the previously generated habit becomes invalid. In this paper, we define the minimum number of navigation episodes required to generate a habit as 1.2 times the number of navigation steps at the 30th time when the environment changes or a detour is required. In the experiment, the number of navigation steps in the 30th time is determined by the hippocampal model, which equal to 61, so the number of navigation episodes needed to form habits is 61 × 1.2 = 73.2. Therefore, starting from the 74th navigation, the prefrontal cortex model began to select the strategy generated by the striatal model for navigation, as shown in Figure 21.

**Figure 22:**
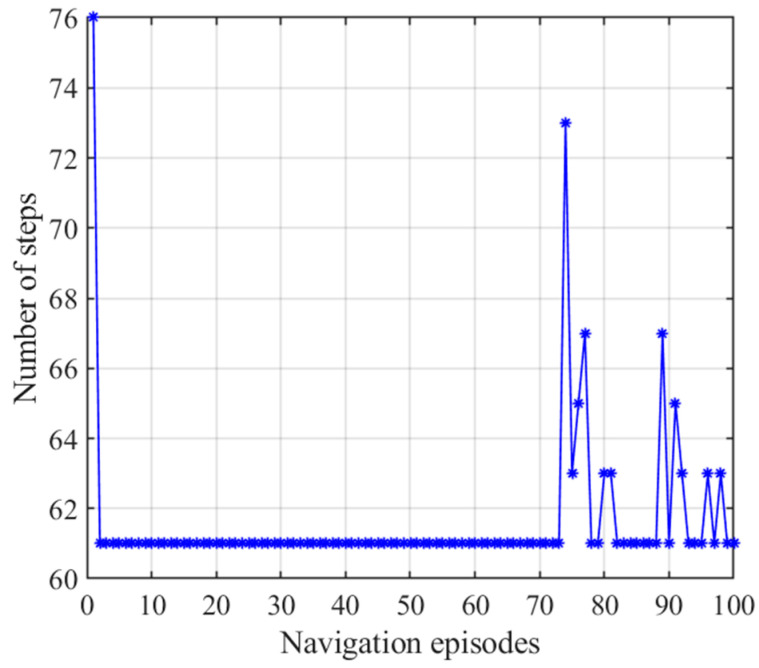
Number of steps with the NLM-HS when door B is closed. Blue asterisks indicate the navigation steps for each navigation episode.

**Figure 23:**
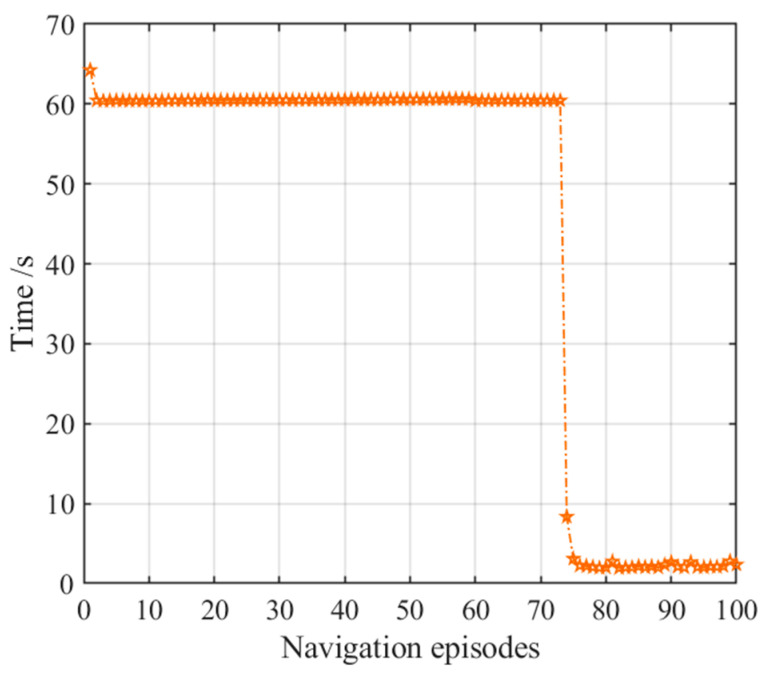
Navigation time with the NLM-HS when door B is closed. Orange pentagrams indicate the navigation time for each navigation episode.

The number of navigation steps and navigation time with the NLM-HS when door B is closed are shown in Figure 22 and Figure 23, respectively, from which we can see that the agent needs less time when using behavioral habits from the striatal model after 73 navigation episodes, while the agent has relatively permanent navigation steps when using the place cell sequence learning algorithm in the hippocampus. The agent navigates using habits before encountering an obstacle, after which the prefrontal cortex model chose to use a strategy in the hippocampal model for navigation. The habit previously generated was completely abandoned, and new habits were generated by interacting with the environment. Furthermore, we do not choose to use the previous habit because the path generated by hippocampal place cell sequence planning directly leans toward the optimal path. If the previously generated habit is used, the agent cannot learn the optimal path generated by the hippocampal model well, and may easily fall into a local minimum. The number of navigation steps fluctuated after 73 navigation episodes because the agent starts to adopt the strategy generated from the striatal model, and the striatal model has a certain exploration rate. However, this shortcoming can be compensated by the low time consumption of the NLM-HS.

### 3.4. Results Comparison and Analysis

The results are compared and analyzed in this section.

**Figure 24:**
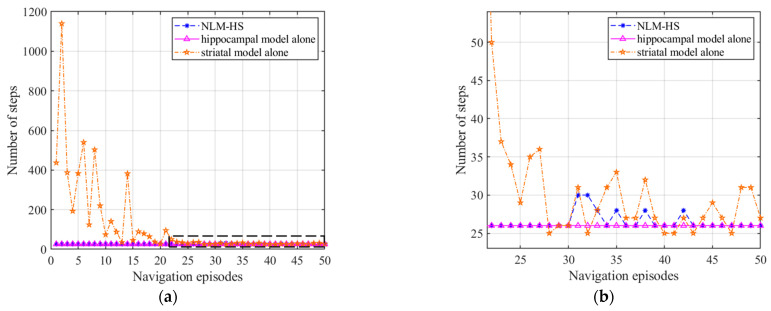
Comparison of navigation steps of the three models. (**a**) Comparison of navigation steps; (**b**) Enlarged version of the dotted box in (**a**).

Figure 24 displays the comparison of navigation steps of the three models, which illustrates that both the hippocampal model alone and the NLM-HS reach the goal with a relatively optimal number of steps at the first navigation, while using the striatal model alone needs more steps to reach the goal. After 30 navigation episodes, the number of navigation steps of the NLM-HS of this model quickly stabilizes after slight fluctuations, while the number of navigation steps of the striatal model alone still fluctuates. Figure 24 also shows the slower learning convergence speed of the striatal model alone. We can conclude that the NLM-HS and the hippocampal model alone are better than the striatal model alone in terms of navigation steps.

**Figure 25:**
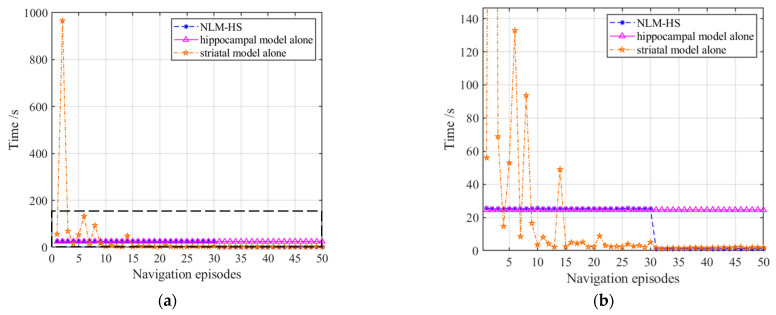
Comparison of navigation time of the three models. (**a**) Comparison of navigation time; (**b**) Enlarged version of the dotted box in (**a**).

Figure 25 displays the comparison of navigation time of the three models, which illustrates that both the hippocampal model alone and the NLM-HS only take less time to reach the goal than the striatal model alone at the first navigation. However, the striatal model alone and the NLM-HS take less time than the hippocampal model alone after stabilization, which benefits from the generation of behavioral habits. We can conclude that the NLM-HS and the striatal model alone are better than the hippocampal model alone in terms of navigation time after stabilization.

In order to increase the reliability of the results and the rationality of the conclusions, we carried out statistical experiments, repeated the above experiments five times, and gave the average values of navigation steps and navigation time for the first time and after stabilization, as shown in Table 3. Table 3 provides the comparison of average navigation results among the three models, and we show the values that performed well in the three models in bold type. In Table 3, when calculating the stabilized navigation steps and time, we take the average of the last five times. The first navigation after door B is closed contains two periods: navigation from the start position to door B and navigation from door B to the goal. The average time of the first navigation in the NLM-HS is lower than that in the hippocampal model because the NLM-HS uses the strategy of habitual navigation in the striatal model from the start position to door B, which saves time. The forward sweep time of the hippocampal model is proportional to the path lengths to the goal. Firstly, the hippocampal model and the NLM-HS can reach the goal with a relatively optimal number of steps not only during the first navigation episode but also when they meet with sudden obstacles, while the striatal model needs considerable time to reach the goal in the same situation, which shows the efficiency and adaptability of the hippocampal model and the NLM-HS in navigation steps. Secondly, we can see that the striatal model and the NLM-HS can eventually converge to a relatively optimal path and only need a slight amount of time to navigate for their usage of habits, which shows the advantage of the striatal model and the NLM-HS in navigation time. Taken together, the NLM-HS demonstrates a better navigation effect than the other two models.

In summary, on the one hand, compared with the continuous and infinite state space in the striatal model alone, the NLM-HS uses the discrete and finite hippocampal cognitive map as input to accelerate the learning convergence speed in the striatal model. On the other hand, compared with the hippocampal model alone, the NLM-HS enables the agent to choose the optimal behavioral decisions faster, by using behavioral habits formed by the striatal model. In addition, the adaptability to changes in surroundings is improved compared with the striatal model alone. When encountering unexpected changes such as obstacles, the agent updates the cognitive map first based on the hippocampal model and then navigates according to the updated cognitive map, thus achieving better adaptivity. Moreover, the NLM-HS elaborates the flexible switching between goal-directed and habitual learning in animal navigation, enabling agents to show environmental cognition and navigation behavior similar to animals.

## 4. Discussion

This paper proposes a NLM-HS, in which the prefrontal cortex arbitration mechanism is designed to arbitrate goal-directed navigation of the hippocampus and habitual navigation of the striatum. In this NLM-HS, the hippocampal and striatal models play a complementary role in different stages of behavioral learning, which is consistent with physiological conclusions [33]. The NLM-HS supports the hypothesis that the prefrontal cortex may serve as a common link between the hippocampus and striatum, which facilitates goal-directed behavior. The navigation cognitive process can be summarized as follows: In initial goal-directed navigation, the hippocampal model provides an initial fast associative memory among the current location, the goal and the environment, and the striatal model has not yet formed a state–action association, so the prefrontal cortex model uses the actions in the hippocampal model. The striatal model forms a state–action association during navigation episodes, and the agent generates behavioral habits gradually, which enables rapid decision making, so the prefrontal cortex model adopts actions in the striatal model later. The behavioral decision-making mechanism of the prefrontal cortex is of great significance to goal-directed navigation. By judging confidence, the prefrontal cortex model can take advantage of both hippocampal and striatal models, arbitrating goal-directed and habitual navigation strategies. Through the combination of hippocampal and striatal models, the agent can achieve a better cognition of the environment, faster decision making and better adaptability in autonomous navigation.

One of the characteristics of the NLM-HS is to apply the cognitive map generated by the hippocampal model to the striatal navigation learning model, which promotes the convergence rate of habit generation. Banquet et al. pointed out that both goal-directed cognitive learning systems and habitual learning systems receive similar hippocampal transition field inputs [34], which shows the same usage of hippocampal cognitive maps. The advantage of using the hippocampal cognitive map as the input for habit generation in the striatum is that the state space that needs to be learned is greatly reduced, thereby accelerating the convergence speed of habit generation in the striatum. The other characteristic of the NLM-HS is staged strategic arbitration in the prefrontal cortex. In contrast to arbitrating every step, the strategic arbitration is more in line with the navigation mechanism of animals. Killcross et al. pointed out that the prefrontal cortex is related to the shift from goal-directed behavior to habitual behavior according to the training being limited or extended [35]. Domenech et al. indicated that the prefrontal executive system alters the functional significance of behavioral events proactively according to the beliefs of the agents about their own behavior, and that the prefrontal cortex resolves the exploitation-exploration dilemma through a two-stage process: a proactive ventromedial stage that constructs the functional significance of upcoming action outcomes, and a reactive dorsomedial stage that guides behavior in response to action outcomes [36]. Consequently, we suppose that the two stages can be related to the hippocampal and striatal systems, with the former performing mainly goal-directed navigation and the latter performing mainly habitual navigation, and the prefrontal cortex model chooses the most appropriate strategies to guide navigation according to confidence in different stages.

As we all know, statistical analysis of a large number of experimental results is an effective guarantee for the correctness of the experimental results and conclusions. During the environmental cognition stage, the agent explores the environment randomly and generates the cognitive map gradually. In this paper, we chose 1000 reachable positions for the generation of cognitive map, and the generation process is shown in Figure 6. In the fixed environment, the number of activated place cells in the whole environment is roughly the same, and it has nothing to do with the increase of reachable positions, which is elaborated in our previous work [30]. During the navigation stage, we compared the basic and adaptive navigation results among the hippocampal model alone, the striatal model alone and the NLM-HS model. The navigation results in the hippocampal model alone are basically the same due to the fixed cognitive map. When using the striatal model alone, due to the randomness of the agent’s choice of actions, the navigation path and time show great differences across the different navigation episodes, which is also the case when the NLM-HS uses striatal strategies. Through statistical experiments, we can conclude that the NLM-HS elaborates the flexible switching between goal-directed and habitual learning, and it has a better navigation effect than the other two models, enabling agents to show environmental cognition and navigation behavior similar to animals.

However, why is the prefrontal cortex model needed to arbitrate between the hippocampal and striatal systems? Although no known direct anatomical connection between the hippocampus and the striatum has been identified in humans, the hippocampus and striatum are anatomically connected by other structures that may support the interaction between episodic memory and behavioral control systems. The prefrontal cortex is thought to be the pathway through which the hippocampus and striatum interact [37,38]. Doeller et al. found that when the hippocampal and striatal systems use the corresponding strategy and both are similarly active, prefrontal cortex activity increases accordingly, indicating the role of the prefrontal cortex in mediating between the two strategies [22]. Although physiological studies have shown that the prefrontal cortex has a mechanism to weigh the effects of the hippocampus and striatum, it is rare to use this mechanism for navigation modeling of agents or robots. In our paper, the prefrontal cortex model implements a competitive choice mechanism between actions from the hippocampal model and striatal model, which can disambiguate decisions that only one of them may find ambiguous.

In addition to the arbitration mechanism, the prefrontal cortex is often combined with the single hippocampus or striatum to complete navigation. For example, Daw et al. suggested that the basal ganglia select behaviors based on a past history of reinforcement, while the prefrontal cortex implements model-based control based on theories or strategies [39]. Some researchers believe that the basal ganglia undergo an inflexible learning process, confined to past experiments, and then interact with flexible representations of the prefrontal cortex [40]. Cazin et al. tested the hypotheses that hippocampal replay allows the prefrontal cortex to eliminate nonoptimal trajectories, thus improving prefrontal cortex learning [41]. The reason for the different effects of the prefrontal cortex model lies in the different speculations and judgments about the function of the prefrontal cortex in physiology; that is, the neural mechanism of the prefrontal cortex in navigation has not yet reached a unified conclusion in physiology. Constructing prefrontal cortex models from different perspectives can not only give the agent a relatively effective cognitive navigation mechanism but also provide insights into the deep research on navigation mechanisms in physiology, which is also the significance of our work.

## 5. Conclusions

In this paper, a navigation learning model based on the hippocampal–striatal circuit (NLM-HS) was established, which provides a possible explanation for the navigation mechanism in the animal brain. The reproduction of classical Tolman detour experiments is provided to demonstrate the reasonability of the model in explaining animal navigation behavior and effectiveness in reproducing animal navigation tasks. This NLM-HS is different from the hippocampal model alone used for goal-directed learning or the striatal model alone used for habitual learning, since the NLM-HS combines them to realize flexible switching between them. The agent learns a cognitive map of the environment gradually by exploring the unknown environment. Then, the agent starts navigation using the arbitration mechanism in the prefrontal cortex model according to confidence, which is one of the main characteristics of the NLM-HS. The major advantage of the NLM-HS is that it can reproduce the process of animal environmental cognition and navigation well, by combining the three brain structures related to navigation and can succeed in explaining how these structures work together to contribute to the whole navigation learning procedure. This study is an exploration of the spatial cognition and navigation of animals, which not only provides a possible explanation of animal navigation mechanisms but can also help to construct intelligent mobile robots with navigation abilities similar to those of humans and animals.

There are also some limitations in the model. For example, the judgment of habit generation is relatively simple. The paper simply uses a certain number of navigation episodes to judge habit generation, which needs to be further designed in detail. In addition, the striatal model used is a classic TD learning model, which needs a substantial amount of time to converge in a large environment and affects the navigation performance of the NLM-HS when using habits. In the future, we intend to further study the refined judgment of habit generation and to study a more efficient striatal model to be used in the NLM-HS for navigation.

## Figures and Tables

**Table 1:** A qualitative analysis of “confidence” in the prefrontal cortex during different periods.

	Initial Period	After Behavioral Habit Is Formed	After Environment Is Changed	After New Behavioral Habit Is Formed
${\mathrm{CONF}}_{\mathrm{HP}}$	${1/R}_{\mathrm{HP}}$	${1/R}_{\mathrm{HP}}$	${1/R}_{\mathrm{HP}}$	${1/R}_{\mathrm{HP}}$
${\mathrm{CONF}}_{\mathrm{Str}}$	0	${1/R}_{\mathrm{Str}}$	0	${1/R}_{\mathrm{Str}}$
${\mathrm{CONF}}_{\mathrm{PFC}}$	>0	<0	>0	<0
Choose strategy from	HP	Str	HP	Str

${\mathrm{CONF}}_{\mathrm{HP}}$, ${\mathrm{CONF}}_{\mathrm{Str}}$ and ${\mathrm{CONF}}_{\mathrm{PFC}}$ represent the “confidence” values of strategies in the hippocampal, striatal, and prefrontal cortex models, respectively. $R_{\mathrm{HP}}$ and $R_{\mathrm{Str}}$ represent the “rapidity” of the hippocampal and striatal models, respectively. HP indicates the hippocampal model. Str indicates the striatal model.

**Table 2:** Integrated navigation algorithm based on the arbitration mechanism in the prefrontal cortex.

Line No.	Integrated Navigation Algorithm
1	Input: agent’s start and goal position, number of iterations $episode_{count}$,
2	Output: navigation strategies at each activated place cell.
3	Initialization: $episode_{count}$; growing threshold $V_{\mathrm{GT}}$; the expected discounted return $Q_{\mathrm{Str}}(s_{t},a_{t})$; Assign start position to current position.
4	For navigation episode $t<episode_{count}$, loop
5	(1) Obtain the activated place cell corresponding to the current position;
6	(2) Choose action.
7	Agent produces strategy in HP according to a place cell sequence planning algorithm.
8	Agent produces strategy in Str according to the improved ε-greedy algorithm in the matrix.
9	Calculate the confidence ${\mathrm{CONF}}_{\mathrm{HP}}$ and ${\mathrm{CONF}}_{\mathrm{Str}}$.
10	Calculate the confidence CONF in the prefrontal cortex;
11	Agent chooses actions according to CONF.
12	(3) Record or update.
13	(3.1) update in HP.
14	if changes in environment are detected, then
15	update the cognitive map according to the dynamic pruning mechanism;
16	end if.
17	Return to (2).
18	(3.2) Update the expected discounted return $Q_{\mathrm{Str}}(s_{t},a_{t})$ in Str.
19	(4) End judgment. If the goal is reached, then end the navigation episode; else, repeat step (2)~(4).
20	End loop

**Table 3:** Comparison of average navigation results among the three models.

Models	HP Model	Str Model	NLM-HS
Average steps of first navigation steps	**26**	517.40	**26**
Average steps of stabilized navigation steps	**26**	27.76	**26.32**
Average time of first navigation time	**24.54 s**	208.01 s	**25.91 s**
Average time of stabilized navigation time	24.54 s	**2.80 s**	**1.83 s**
Average steps of first navigation after door B is closed	**76**	1378.6	**76**
Average steps of stabilized navigation after door B is closed	**61**	85.68	**61.48**
Average time of first navigation after door B is closed	**74.99 s**	777.82 s	**64.25 s**
Average time of stabilized navigation after door B is closed	60.30 s	**12.76 s**	**3.63 s**

Bold represents the values that performed well in the three models. Since the results of this table are all statistical values, some navigation steps may not be integers.

## Data Availability

Data is contained within the article.

## References

[1] Ni J., Wu L., Fan X., Yang S.X. (2016). Bioinspired intelligent algorithm and its applications for mobile robot control. Comput. Intel. Neurosc..

[2] Barrera A., Weitzenfeld A. (2008). Biologically-inspired Robot Spatial Cognition based on Rat Neurophysiological Studies. Auton. Robot..

[3] Wyeth G., Milford M. (2009). Spatial cognition for robots. IEEE Robot. Autom. Mag..

[4] Geva-Sagiv M., Las L., Yovel Y., Ulanovsky N. (2015). Spatial cognition in bats and rats: From sensory acquisition to multiscale maps and navigation. Nat. Rev. Neurosci..

[5] Epstein R.A., Patai E.Z., Julian J.B., Spiers H.J. (2017). The cognitive map in humans: Spatial navigation and beyond. Nat. Neurosci..

[6] Maffeia G., Santos-Pataa D., Marcosa E., Sánchez-Fiblaa M., Verschure P.F.M.J. (2015). An embodied biologically constrained model of foraging: From classical and operant conditioning to adaptive real-world behavior in DAC-X. Neural Netw..

[7] Pennartz C.M.A., Ito R., Verschure P.F.M.J., Battaglia F.P., Robbins T.W. (2011). The hippocampal-striatal axis in learning, prediction and goal-directed behavior. Trends Neurosci..

[8] Chersi F., Pezzulo G. (2012). Using hippocampal-striatal loops for spatial navigation and goal-directed decision-making. Cogn. Process..

[9] Peer M., Brunec I.K., Newcombe N.S., Epstein R.A. (2020). Structuring Knowledge with Cognitive Maps and Cognitive Graphs. Trends Cogn. Sci..

[10] Tolman E.C. (1948). Cognitive maps in rats and men. Psychol. Rev..

[11] O’Keefe J., Dostrovsky J. (1971). The hippocampus as a spatial map. Preliminary evidence from unit activity in the freely-moving rat. Brain Res..

[12] Mehta M.R. (2015). From synaptic plasticity to spatial maps and sequence learning. Hippocampus.

[13] Pfeiffer B.E., Foster D.J. (2013). Hippocampal place-cell sequences depict future paths to remembered goals. Nature.

[14] Milford M. (2014). Principles of goal-directed spatial robot navigation in biomimetic models. Philos. Trans. R. Soc. B.

[15] Babayan B.M., Watilliaux A., Viejo G., Paradis A.L., Girard B., Rondi-Reig L. (2017). A hippocampo-cerebellar centred network for the learning and execution of sequence-based navigation. Sci. Rep..

[16] Lansink C.S., Goltstein P.M., Lankelma J.V., McNaughton B.L., Pennartz C.M.A. (2009). Hippocampus leads ventral striatum in replay of place-reward information. PLoS Biol..

[17] Moussa R., Poucet B., Amalric M., Sargolini F. (2011). Contributions of dorsal striatal subregions to spatial alternation behavior. Learn. Mem..

[18] Ashby F.G., Turner B.O., Horvitz J.C. (2010). Cortical and basal ganglia contributions to habit learning and automaticity. Trends Cogn. Sci..

[19] Bornstein A.M., Daw N.D. (2011). Multiplicity of control in the basal ganglia: Computational roles of striatal subregions. Curr. Opin. Neurobiol..

[20] Maniadakis M., Trahanias P., Tani J. (2012). Self-organizing high-order cognitive functions in artificial agents: Implications for possible prefrontal cortex mechanisms. Neural Netw..

[21] Srinivasa N., Chelian S.E. (2012). Executive control of cognitive agents using a biologically inspired model architecture of the prefrontal cortex. Biol. Inspir. Cogn. Arc..

[22] Doeller C.F., King J.A., Burgess N. (2008). Parallel striatal and hippocampal systems for landmarks and boundaries in spatial memory. Proc. Natl. Acad. Sci. USA.

[23] Barraclough D.J., Conroy M.L., Lee D. (2004). Prefrontal cortex and decision making in a mixed-strategy game. Nat. Neurosci..

[24] Stachenfeld K.L., Botvinick M.M., Gershman S.J. (2017). The hippocampus as a predictive map. Nat. Neurosci..

[25] Yu N., Zhai Y., Yuan Y., Wang Z. (2019). A Bionic Robot Navigation Algorithm Based on Cognitive Mechanism of Hippocampus. IEEE Trans. Autom. Sci. Eng..

[26] Zhao F., Zeng Y., Wang G., Bai J., Xu B. (2018). A Brain-Inspired Decision Making Model Based on Top-Down Biasing of Prefrontal Cortex to Basal Ganglia and Its Application in Autonomous UAV Explorations. Cogn. Comput..

[27] McDonald R.J., Hong N.S., Devan B.D. (2005). The challenges of understanding mammalian cognition and memory-based behaviours: An interactive learning and memory systems approach. Neurosci. Biobehav. Rev..

[28] Pezzulo G., Rigoli F., Chersi F. (2013). The Mixed Instrumental Controller: Using Value of Information to Combine Habitual Choice and Mental Simulation. Front. Psychol..

[29] Chersi F., Burgess N. (2015). The Cognitive Architecture of Spatial Navigation: Hippocampal and Striatal Contributions. Neuron.

[30] Ruan X., Chai J., Wu Y., Zhang X., Huang J. (2021). Cognitive map construction and navigation based on hippocampal place cells. Acta Autom. Sin..

[31] Lee M.G., Jun G., Choi H.S., Jang H.S., Bae Y.C., Suk K., Jang I.S., Choi B.J. (2010). Operant conditioning of rat navigation using electrical stimulation for directional cues and rewards. Behav. Process.

[32] Alvernhe A., Save E., Poucet B. (2011). Local remapping of place cell firing in the Tolman detour task. Eur. J. Neurosci..

[33] Brown T.I., Ross R.S., Tobyne S.M., Stern C.E. (2012). Cooperative interactions between hippocampal and striatal systems support flexible navigation. Neuroimage.

[34] Banquet J.P., Hanoune S., Gaussier P., Quoy M., Villa A., Masulli P., Pons Rivero A. (2016). From Cognitive to Habit Behavior during Navigation, through Cortical-Basal Ganglia Loops. Artificial Neural Networks and Machine Learning—ICANN 2016.

[35] Killcross S., Coutureau E. (2003). Coordination of Actions and Habits in the Medial Prefrontal Cortex of Rats. Cereb. Cortex.

[36] Domenech P., Rheims S., Koechlin E. (2020). Neural mechanisms resolving exploitation-exploration dilemmas in the medial prefrontal cortex. Science.

[37] Graham S., Phua E., Soon C.S., Oh T., Au C., Shuter B., Wang S.C., Yeh I.B. (2009). Role of medial cortical, hippocampal and striatal interactions during cognitive set-shifting. Neuroimage.

[38] Dahmani L., Bohbot V.D. (2015). Dissociable contributions of the prefrontal cortex to hippocampus- and caudate nucleus-dependent virtual navigation strategies. Neurobiol. Learn. Mem..

[39] Daw N.D., Niv Y., Dayan P. (2005). Uncertainty-based competition between prefrontal and dorsolateral striatal systems for behavioral control. Nat. Neurosci..

[40] Seger C.A., Spiering B.J. (2011). A Critical Review of Habit Learning and the Basal Ganglia. Front. Syst. Neurosci..

[41] Cazin N., Alonso M.L., Chiodi P.S., Pelc T., Harland B., Weitzenfeld A., Fellous J.M., Dominey P.F. (2019). Reservoir computing model of prefrontal cortex creates novel combinations of previous navigation sequences from hippocampal place-cell replay with spatial reward propagation. PLoS Comput. Biol..

